# MEKK1 Regulates Chemokine Expression in Mammary Fibroblasts: Implications for the Breast Tumor Microenvironment

**DOI:** 10.3389/fonc.2021.609918

**Published:** 2021-03-18

**Authors:** Saverio Gentile, Najmeh Eskandari, Michael A. Rieger, Bruce D. Cuevas

**Affiliations:** ^1^ Division of Hematology Oncology, Department of Medicine, University of Illinois Chicago, Chicago, IL, United States; ^2^ Molecular Neurobiology Laboratory, Salk Institute for Biological Studies, La Jolla, CA, United States; ^3^ Division of Basic Biomedical Sciences, Sanford School of Medicine, University of South Dakota, Vermillion, SD, United States

**Keywords:** MEKK1, chemokine, gene expression, kinase, breast cancer, tumor microenvironment

## Abstract

Breast tumors contain both transformed epithelial cells and non-transformed stroma cells producing secreted factors that can promote metastasis. Previously, we demonstrated that the kinase MEKK1 regulates cell migration and gene expression, and that transgene-induced breast tumor metastasis is markedly inhibited in MEKK1-deficient mice. In this report, we examined the role of MEKK1 in stroma cell gene expression and the consequent effect on breast tumor cell function. Using a heterotypic cell system to quantify the effect of stroma cells on breast tumor cell function, we discovered that MEKK1−/− fibroblasts are significantly less effective at inducing tumor cell invasion than MEKK1+/+ fibroblasts. Expression array analysis revealed that both baseline and tumor cell-induced expression of the chemokines CCL3, CCL4, and CCL5 were markedly reduced in MEKK1−/− mammary fibroblasts. By focusing on the role of MEKK1 in CCL5 regulation, we discovered that MEKK1 kinase activity promotes CCL5 expression, and inactive mutant MEKK1 strongly inhibits CCL5 transcription. CCL5 and the other MEKK1-dependent chemokines are ligands for the GPCR CCR5, and we show that the CCR5 antagonist Maraviroc strongly inhibits fibroblast-induced tumor cell migration. Finally, we report that fibroblast growth factor 5 (FGF-5) is secreted by MDA-MB 231 cells, that FGF-5 activates MEKK1 effectors ERK1/2 and NFκB in fibroblasts, and that chemical inhibition of NFκB inhibits CCL5 expression. Our results suggest that MEKK1 contributes to the formation of a breast tumor microenvironment that supports metastasis by promoting expression of stroma cell chemokine genes in response to tumor cell-induced paracrine signaling.

## Introduction

In addition to transformed mammary epithelial cells, breast tumors consist of an array of non-transformed stroma cells that interact with cancer cells and infiltrate the tumor mass (1). Evidence compiled from gene expression studies (2), creative *in vivo* imaging experiments (3) and 3D culture approaches (4) to recreate the tumor environment support a model wherein stroma cells influence tumor cell function. The possibility that this diverse cellular milieu might influence tumor progression and response to therapy has spurred considerable interest in the role of breast stroma cells in the tumor environment. Some reported examples include cancer-associated fibroblasts (CAFs) that promote tumor cell growth and metastasis (5), and tumor-associated macrophages forming a paracrine “pas de deux” with cancer cells to provoke mutual migration (6). These examples and others show that stroma cells can promote metastasis through the expression and secretion of factors such as cytokines that bind receptors on tumor cells to induce functional responses ranging from proliferation to cell migration. The CC class of chemokines (7) are known to induce immune cell chemotaxis, and CAFs have been shown to express CCL proteins (1). Indeed, several lines of evidence support a role for CCL chemokines in tumor progression. For example, Silzle and colleagues demonstrated that CAFs produce CCL2 and the pro-inflammatory cytokine IL-6, and CAF-induced monocyte recruitment in a co-culture system required CCL2 (8). In addition, both CAFs harvested from breast tumors and normal renal fibroblasts have been found to express CCL5/RANTES (9); (10), and CCL5 has been found to be highly expressed in aggressive breast tumors (11–14). Largely characterized in the regulation of T cell and monocyte function in immune response (15), CCL5 induces chemotaxis when bound by its high affinity receptors, including the seven transmembrane G protein-coupled receptor CCR5 (16). Interestingly, some tumor cells express chemokine receptors as well, suggesting that stromal fibroblast chemokine production can influence the tumor microenvironment and promote tumor cell migration and invasion (12, 14). CCL5 can induce breast tumor cell migration through binding and activation of CCR5, and an inhibitory mutant CCL5 (met-RANTES) inhibits breast tumor growth and migration (17). In addition to CCL5, CCR5 has been shown to bind multiple chemokine ligands, including CCL3 and CCL4 (16). Overall, these reports suggest that this nexus of tumor cells that express CCR5 and stroma cell-derived CC class chemokine ligands provide breast cancer investigators a new set of novel therapeutic targets (7, 12, 13, 16, 18, 19). Due to the role of CCR5 as a key factor in HIV infection, CCR5 inhibitors such as Maraviroc/UK-427,857 have been developed and approved for use as anti-retroviral therapy in HIV patients (20). Taken together, studies showing that CCR5 ligands may be highly expressed in breast tumors suggests that therapeutics designed to prevent viral infection of T cells may be useful if repurposed as cancer drugs.

The current understanding of the transcriptional regulatory mechanisms that control CCL expression is less developed than that of CCR5 function. Transcription of CCL5 expression is at least partly under control of transcription factor response elements within the 5’ promoter region that are bound by AP-1 and NFκB transcription factor proteins (21, 22). AP-1 (activating protein 1) dimeric transcription factors control gene expression in response to a formidable array of environmental stimuli, and are regulated by both phosphorylation and ubiquitylation (23–26). NFκB also is regulated by upstream kinase activity that is required to relieve inhibition by regulatory proteins (27–29). AP-1 regulators include the family of mitogen-activated protein kinases (MAPK), with multiple isoforms of the ERK, JNK, and p38 proteins that have long been established as AP-1 regulators that both directly phosphorylate AP-1 proteins and indirectly control AP-1 function by activating MAPK-dependent kinases that consequently phosphorylate AP-1 proteins (24, 30, 31). MAPK activity is tightly controlled by a complex network that mediates MAPK phosphorylation, dephosphorylation and ubiquitylation, but at the core of MAPK control is the consecutive activation of three kinases that form the canonical MAPK cascade, consisting of a MAPK that is phosphorylated and activated by a MAPK kinase (MAP2K) that is, in turn, phosphorylated and activated by a MAPK kinase kinase (MAP3K) (30). MEKK1 is a MAP3K protein that our group and others have shown the MAP3K MEKK1 to be a key regulator of both ERK1/2 and JNK MAPK activity (30–32). MEKK1 activity promotes AP-1 phosphorylation and activation (24, 30), and also has been shown to phosphorylate and activate IκB kinases, leading to NFκB activation (27–29, 33). Furthermore, our group and others have demonstrated that MEKK1 further controls AP-1 activity through control protein stability (24–26), thus identifying MEKK1 as a key regulator of both AP-1-dependent and NFκB-dependent gene transcription and expression.

Our earlier work revealed that MEKK1-deficient mice are resistant to transgene-induced breast tumor metastasis (34), and that MEKK1 is necessary for expression of cancer cell proteases that are associated with breast tumor cell invasiveness, but did not define the role of stroma cells in these breast tumors. In the present study, we model the breast tumor microenvironment by exposing mammary fibroblasts to soluble factors derived from breast tumor cells, and then perform expression array analysis of mammary fibroblasts, comparing cells that express MEKK1 to MEKK1-decient cells. We reveal that MEKK1 regulates chemokine expression in mammary fibroblasts that induces breast tumor cell migration. Our results indicate that MEKK1 controls metastasis-related functions of both cancer cells and non-cancer stroma cells, and thereby plays a key role in determining the nature of the breast tumor cellular environment that influences tumor metastasis.

## Materials and Methods

### Antibodies and Reagents

Antibodies specific for MEKK1 were purchased from Santa Cruz Biotechnology. Anti-pERK antibodies were purchased from Cell Signaling Technology. U0126 was purchased from Promega (Madison, WI), CAY10512 was purchased from Cayman Chemical (Ann Arbor, MI), SSR128129E was purchased from Selleckchem (Houston, TX), Maraviroc, and PEITC were purchased from Sigma-Aldrich (St. Louis, MO).

### Cell Culture and Transfection

MDA-MB 231 breast tumor cells, HTB-125 human mammary fibroblast cells and primary mouse mammary fibroblasts were cultured in DMEM (Dulbecco’s modified Eagle’s medium) (Invitrogen) containing 10% (v/v) fetal bovine serum (Atlanta Biologicals) at 37°C and maintained in 5% CO_2_ in a humidified atmosphere. All transfections were conducted using linear polyethylenimine (PEI, MW 25000, Polysciences Inc., Warrington, PA). Briefly, PEI was dissolved in water and mixed with DNA vectors at a 3:1 ratio (µg/µg) in OptiMEM (Life Technologies) and allowed to form complexes for 20 min at room temperature prior to adding the complexed DNA dropwise to cells in culture. Cells were harvested 48 h post-transfection.

### Isolating Primary Mammary Fibroblasts

The inguinal mammary glands from 8-10 week-old female mice were removed, minced to <1mm pieces and then mixed with serum-free DMEM. The tissue was digested at 37C with shaking in 2.5 mg/ml collagenase type 3 (Worthington), 1 mg/ml hyaluronidase (Sigma) and 100 U/ml pen/strep (Life Technologies) for 3 h. The digestion mixture was then allowed to rest at room temperature for 10 min to allow the fat to collect at the top of the fluid and an epithelial cell pellet to form at the bottom of the tube. The fat is then removed and the cell suspension is transferred to a new sterile centrifuge tube. The pellet is discarded. The cell suspension is centrifuged at 800 × g for 10 min, and the fluid discarded. The pellet containing the fibroblasts is re-suspended in DMEM with 10% serum and transferred to 10 cm tissue culture plates.

### RNA Extraction and Expression Arrays

Total cell RNA was extracted with the RNeasy kit (Qiagen, Valencia, CA, USA). Reverse transcription, cDNA synthesis and expression array hybridization, chip reading and preliminary analysis of the array results were performed by the Northwestern University Genomics CORE facility. An Illumina Mouse 8 chip platform was utilized to compare expression of mammary fibroblasts derived from six mice (3 × MEKK1+/+, 3 × MEKK1−/−), whereas an Illumina Human chip was utilized to determine gene expression in three separate replicates of MDA-MB 231 cells. Array expression data were z-score normalized and statistical inference was performed according to Cheadle et al. (35). Heat map image representing normalized expression data was generated in MATLAB.

### Immunoblotting

Proteins were separated by SDS-PAGE and transferred on to Protran nitrocellulose membranes (Whatman). Membranes were blocked in 5% (w/v) non-fat dried skimmed milk powder diluted in TBST (20 mM Tris, 137 mM NaCl and 0.1% Tween-20, adjusted to pH 7.6) or 5% globulin-free BSA (Sigma) in TBST and incubated in the appropriate antibody at 4°C overnight. After extensive washing, the membranes were then incubated with HRP (horseradish peroxidase)–conjugated donkey anti-rabbit IgG (Jackson ImmunoResearch) or HRP–sheep anti-mouse IgG (Amersham-GE Healthcare) secondary antibodies for 1 h at room temperature. After extensive washing, the targeted proteins were detected by ECL (enhanced chemiluminescence, Thermo Scientific). Where indicated, blots were stripped by treatment with 2% (w/v) SDS and 100 mM 2-mercaptoethanol in TBS, and then re-probed with the desired antibodies.

### CCL5 Reporter Assay

The Dual-Luciferase Reporter Assay System reagent kit was purchased from Promega (Madison, WI) and reporter assays using transfected cell lysates were performed as per manufacturer recommendations. The pGL2-CCL5-220 reporter plasmid was a generous gift of Dr. Antonella Casola (University of Texas Medical Branch at Galveston, Galveston, TX). Fibroblasts transfected with reporter vectors were exposed to either breast tumor cell-conditioned media or regular growth media +/- indicated drugs for indicated times prior to lysis and quantification of reporter activity.

### CCL5 ELISA

HTB-125 human breast fibroblast cells were treated with MDA-MB-231-conditioned medium for 1, 2 and 4 h, after which the conditioned medium was replaced with regular growth medium for 24 h. The culture supernatants were then collected and any cells removed by filtration. CCL5 concentration was determined using the ELISA for human CCL5/RANTES Kit (R&D Systems Inc. Cat# DY278-05) according to the manufacturer’s protocol. Briefly, 300 µl of reagent diluent were placed in the capture antibody-coated wells to block plate for 1 h. After washing, 100 µl of test samples or standards were added per well and incubated 2 h. The wells were then washed and incubated with 100 µl of detection antibody for 2 h. After washing, 100 µl of the working dilution of streptavidin-HRP was added to each well for 20 min. Following wash step, 100 µl of substrate solution was added before incubation for 20 min. The color reaction was stopped by adding 50 µl of stop solution and the absorbance of the samples was measured using an ELISA plate reader. The CCL5 concentrations were calculated based on the standard curve.

### Breast Cancer Cell Migration Assay

MDA-MB 231 cells were suspended in serum-free medium containing either CCR5 inhibitor Maraviroc (10 µM) or DMSO. Chemotaxis was assessed by Transwell (Corning) assay, wherein 10^5^ cells were allowed to migrate through a filter (8 µm pore) toward 4% serum for 5 h, and then migrating cells were stained with Wright’s stain and counted.

## Results

### Primary Mammary Fibroblasts Induce MEKK1-Dependent Chemotaxis in MDA-MB 231 Breast Tumor Cells

**Figure 1:**
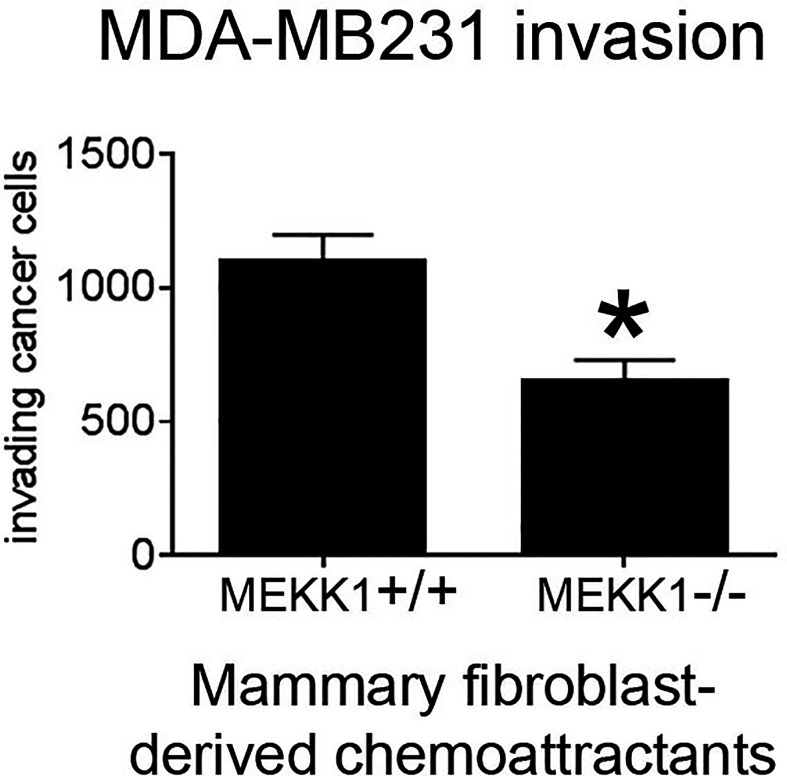
MEKK1 expression is necessary for mammary fibroblasts to promote breast tumor cell invasion. The graph depicts results from BioCoat Matrigel assays of MDA-MB 231 cell invasiveness induced by media that had been conditioned by either MEKK1+/+ or MEKK1−/− mammary fibroblasts for 72 h. The data represented in graph are derived from at least three independent experiments. *p < 0.05 by paired t-test.

We previously reported that transgene-induced breast tumor metastasis is significantly delayed in MEKK1-deficient mice, and the tumors markedly less invasive (34). Breast tumors arising in the ductal lumen are surrounded by basement membrane and assorted stroma cells, including mammary fibroblasts capable of secreting factors that promote tumor cell invasion. To determine whether MEKK1 is required for mammary fibroblasts to induce tumor cell invasiveness, we isolated primary fibroblasts from the mammary glands of both MEKK1+/+ and MEKK1−/− mice and used these cells to condition media for use as an invasion assay chemoattractant. We found that media conditioned by mammary fibroblasts consistently induced MDA-MB 231 breast cancer cell invasion in an *in vitro* assay (**Figure 1**). However, tumor cell invasiveness was significantly diminished when media conditioned by MEKK1-deficient fibroblasts is utilized as the chemoattractant (**Figure 1**). Since direct contact with fibroblasts was not required to induce invasion, these results indicate that the one or more fibroblast-derived factors capable of promoting breast tumor invasiveness is under the control of MEKK1. In order to identify the MEKK1-dependent secreted proteins, we investigated gene expression in mammary fibroblasts.

### Breast Tumor Cells Induce MEKK1-Dependent Chemokine Expression in Mammary Fibroblasts

**Figure 2:**
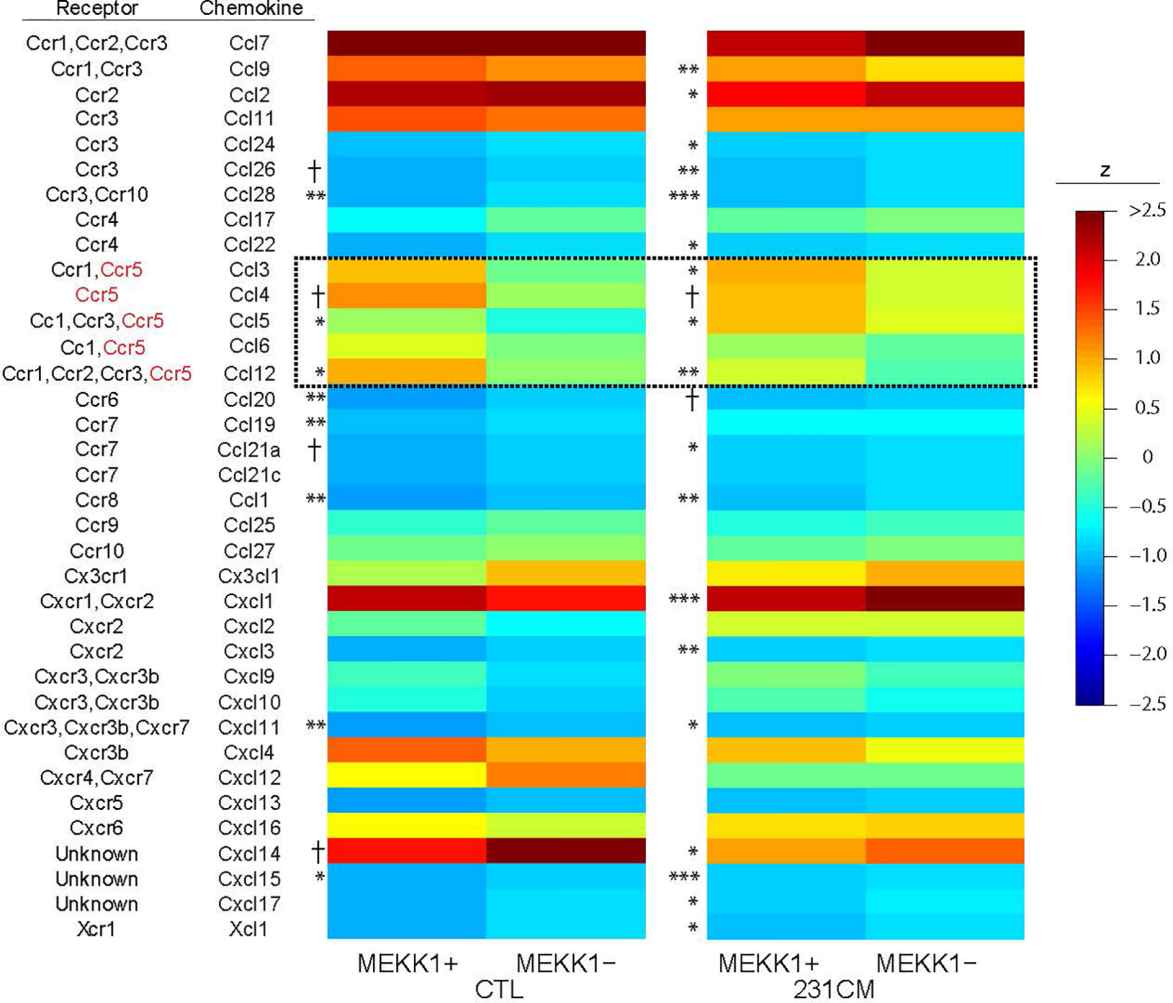
CCL5 expression in primary mammary fibroblasts is enhanced by MEKK1. Heat map of z-score normalized microarray expression data for 36 chemokines in primary mammary fibroblasts cultured from six mice (3× MEKK1^+/+^ and 3× MEKK1−/−) either unstimulated (CTL) or induced with cultured medium from MDA-MB-231 cells (231CM). Columns show mean expression by group (N=4 culture replicates per condition), with asterisks indicating significance by z-tests with Benjamini-Hochberg false discovery rate adjustment for multiple testing. *FDR < 0.05, **FDR <0.01, ***FDR < 0.001, ^†^unadjusted p < 0.05.

To identify MEKK1-dependent secreted factors that could induce breast tumor cell migration and invasion, we performed array analysis comparing gene expression in mammary fibroblasts harvested from both MEKK1+/+ (n=3) and MEKK1−/− mice (n=3). To model fibroblast gene expression induced by the secretory breast tumor environment, we exposed fibroblasts to media conditioned by MDA-MB 231 cells. We focused on identifying MEKK1-dependent genes that encode soluble factors known to promote cell migration, and we discovered that expression of several CC class chemokines displayed reduced expression in MEKK1-deficient fibroblasts (**Figure 2**). Intriguingly, several chemokines that are ligands for the receptor CCR5 (murine CCL3, 4, 5, 6, and 12) displayed reduced expression in MEKK1-deficient cells in either basal conditions, induced conditions, or both. CCL5 expression in particular was reduced under both basal and induced conditions in MEKK1-deficient cells compared to fibroblasts that express MEKK1 (**Figure 2**, basal conditions: 3.16-fold reduction, p=0.017, induced conditions: 2.8-fold reduction, p=0.029). Although reduced in MEKK1 knockout cells, CCL5 expression was induced significantly by breast tumor cell-conditioned media in both MEKK1+/+ and MEKK1−/− fibroblasts, indicating that control of induced CCL5 expression is at least partly independent of MEKK1.

**Figure 3:**
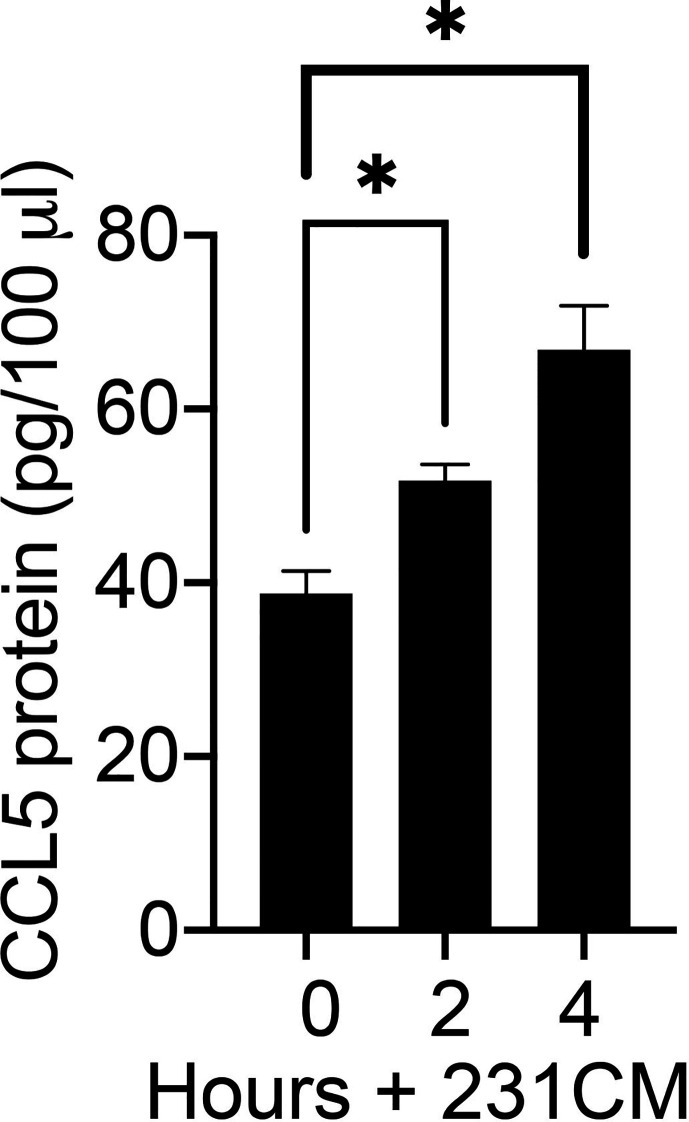
Breast tumor cells stimulate CCL5 protein production in human mammary fibroblasts. The graph depicts results from CCL5 ELISA analysis of cell culture supernatant from HTB-125 human mammary fibroblasts exposed to 231CM compared to supernatant from fibroblasts maintained in regular growth media. Fibroblasts exposed to tumor cell-conditioned media were treated for 2 h or 4 h, after which the media was replaced growth media. 24 h later the media were collected and subjected to ELISA analysis. The data represented in graph are derived from at least three independent experiments. *p < 0.05 against control by t-test.

To determine whether breast tumor-derived soluble factors can induce CCL5 protein expression in human mammary fibroblasts, we measured the CCL5 protein produced by HTB-125 human mammary fibroblasts exposed to MDA-MB 231 cell-conditioned media (**Figure 3**). We observed that CCL5 protein production was significantly increased in human fibroblasts exposed to breast tumor cell factors compared to untreated fibroblasts. Furthermore, we observed that fibroblasts that were exposed to tumor cell-conditioned media for 4 h produced significantly more CCL5 protein than fibroblasts exposed to tumor cell factors for 2 h, suggesting that the magnitude of the fibroblast CCL5 protein production is dose-dependent. Together, our mRNA and protein data indicate that soluble factors produced by breast tumor cells can induce CCL5 protein production in non-tumor mammary stroma cells. Given the reported role of CCL5 in tumor metastasis, we then directed our investigation to define the mechanisms by which MEKK1 controls CCL5 expression.

### CCL5 Expression Requires MEKK1 Kinase Activity, but Not Ubiquitin Ligase Activity

**Figure 4:**
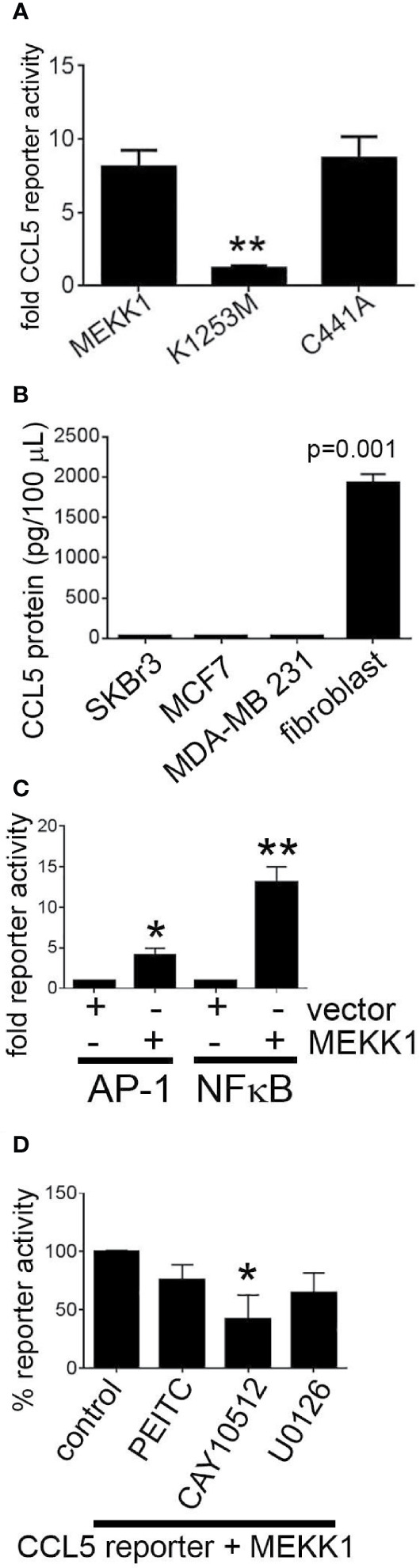
MEKK1 kinase activity, but not ubiquitin ligase activity, is required to induce CCL5 expression. Gene reporter and ELISA analysis are used to determine whether transfection of kinase-inactive mutant MEKK1 (K1253M) or ubiquitin ligase-inactive mutant MEKK1 (C441A) induces CCL5 expression. **(A, D)** Graphs depict CCL5 reporter analysis of mammary fibroblasts transfected with the pGL2-CCL5-220 luciferase reporter vector and a pCDNA3.1/MEKK1 expression vector. **p < 0.01 by t-test. **(B)** CCL5 ELISA analysis of cell culture supernatant from both multiple breast tumor cell lines compared to cell culture supernatant of cancer-associated mammary fibroblasts. **(C)** Graph shows both AP-1 and NFκB reporter analysis of mammary fibroblasts transfected with either the pGL2-AP-1 or pGL2- NFκB luciferase reporter vector and a pCDNA3.1/MEKK1 expression vector or pCDNA3.1 empty vector. **(D)** CCL5 reporter assay analysis showing effect of chemical inhibitors on CCL5 reporter activity. Cells were treated for six hours prior to lysis with the following drugs concentrations: (MEK inhibitor, 10 ng/ml, n = 3), CAY10512 (NFκB inhibitor, 1.3 μg/ml, n = 3), and PEITC (0.3 μM, n = 3). *p < 0.05, **p < 0.01 by t-test.

MEKK1 is unique among kinases in that it contains ubiquitin ligase activity (25, 36). We previously reported that MEKK1 kinase activity was a key component in the regulation of AP-1 transcription factor activity and function (24), and we demonstrated that MEKK1 ubiquitin ligase activity plays a role in transcription factor ubiquitylation and stability (25). MEKK1 phosphorylates the kinases MEK4 and MEK1/2, thereby activating MAPK proteins JNK and ERK1/2 (30). MEKK1 also phosphorylates and activates IKKα and IKKβ, promoting phosphorylation and subsequent degradation of NFκB inhibitor protein IKB and the consequent activation of NFκB (33). To determine whether either MEKK1 kinase activity or ubiquitin ligase activity is necessary for CCL5 expression, we transfected mammary fibroblasts with vectors encoding either wild type MEKK1, kinase-inactive mutant MEKK1 (K1253M), or ubiquitin ligase-inactive mutant MEKK1 (C441A) (36), and co-transfected with a CCL5 luciferase-based reporter vector. In our assay, luciferase expression is driven by the proximal 200 bases of the Ccl5 promoter that is reported to contain both NFκB and AP-1 transcription factor response elements (37). Our reporter assays revealed that CCL5 reporter activity was markedly induced by co-expression of wild type or ubiquitin ligase-mutant MEKK1, whereas kinase inactive mutant MEKK1 did not induce CCL5 promoter activity (**Figure 4A**), indicating that kinase activity is necessary for MEKK1 to promote CCL5 expression. In order to compare the relative CCL5 production of cancer and stroma cells, we utilized ELISA analysis to quantify CCL5 protein secreted by both breast cancer cell lines and mammary fibroblasts that had been harvested from murine mammary tumors (cancer-associate fibroblasts, (34)). To determine whether CCL5 secretion varies among breast cancer cells, we tested media conditioned by breast cancer cell lines that are commonly used to model distinct breast cancer types, including luminal (MCF7), HER2+ (SKBr3), and triple negative (MDA-MB 231) breast cancers. Strikingly, we found that cancer-associated fibroblasts secreted greater than 60-fold more CCL5 protein than any of the breast cancer cells (**Figure 4B**).

To determine how MEKK1 kinase activity regulates CCL5 transcription, we first confirmed the effect of MEKK1 on activation of the AP-1 and NFκB transcription factors. We observed that co-expression of MEKK1 significantly enhanced activity of gene reporters driven by either AP-1 or NFκB response elements (**Figure 4C**). We then sought to elucidate which MEKK1-dependent signaling pathway induces CCL5 expression by including chemical inhibitors of either NFκB or AP-1 transcription in our reporter experiments. We observed that blockade of NFκB by the inhibitor CAY10512 resulted in a 60% reduction in CCL5 reporter activity (p=0.037) (**Figure 4D**), whereas inhibition of ERK activation by MEK blocker U0126 reduced CCL5 reporter activity by 35% (p=0.068). Selective MEKK1 inhibitors have not yet been reported, so to determine whether chemical inhibition of MEKK1 blocks CCL5 reporter activity, we treated transfected fibroblasts with phenethyl isothiocyanate (PEITC), a naturally occurring compound that has been reported to display some inhibitory activity toward MEKK1 (38). We observed that exposure to PEITC consistently, albeit not significantly (p=0.08) reduced CCL5 reporter activity by 24%. These results support the conclusion that MEKK1 kinase activity promotes activation of transcription factors that drive CCL5 expression. Taken together, these data suggest that signaling arising from MEKK1 kinase activity promotes induction of CCL5 expression in mammary fibroblasts, whereas MEKK1 ubiquitin ligase activity is dispensable for CCL5 induction.

### CCR5 Inhibitor Maraviroc Blocks Mammary Fibroblast-Induced Breast Cancer Cell Chemotaxis

**Figure 5:**
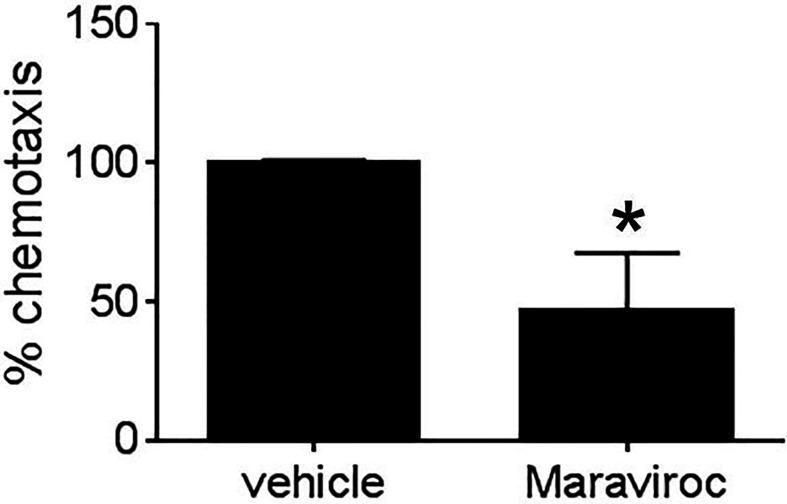
Mammary fibroblast-induced breast tumor cell migration is blocked by CCR5 inhibitor Maraviroc. Graph shows results from Boyden chamber assays of MDA-MB231 cell chemotaxis. Mammary fibroblast-conditioned media was used as a chemoattractant and includes either Maraviroc (10µM) or an equal volume of vehicle. The data represented in graphs are derived from at least three independent experiments. *p < 0.05 against control by t-test.

Given that MEKK1-deficient fibroblasts showed both reduced CCL5 expression (**Figures 2** and **4**) and diminished ability to induce tumor cell invasion (**Figure 1**), we decided to examine the importance of breast stroma-derived CCL5 in MDA-MB 231 tumor cell migration. Several MEKK1-dependent chemokines are CCR5 ligands, and recent reports suggest that CCR5 function may be important in breast cancer metastasis (11, 12, 14, 19). To determine whether CCR5 blockade would inhibit tumor cell migration, we performed chemotaxis assays in the presence of Maraviroc, which is a selective and non-competitive CCR5 antagonist that blocks ligand binding and subsequent signaling (20). We observed that exposure to Maraviroc reduces MDA-MB 231 chemotaxis induced by mammary fibroblast-conditioned media by 50% (**Figure 5**), thus indicating that CCR5 function contributes to tumor cell chemotaxis induced by mammary fibroblasts. When considered with our expression data, these results demonstrate that MEKK1 strongly enhances the ability of mammary fibroblasts to produce chemoattractant proteins that are detected by breast tumor cells. Furthermore, our findings suggest that MEKK1 is a key regulator of CCL5 expression. Once released by mammary fibroblasts, MEKK1-dependent chemokine expression would be predicted to drive migration of CCR5-expressing cells in the breast tumor microenvironment.

### Breast Tumor Cell-Derived FGF-5 Induces CCL5 Expression in Mammary Fibroblasts

**Figure 6:**
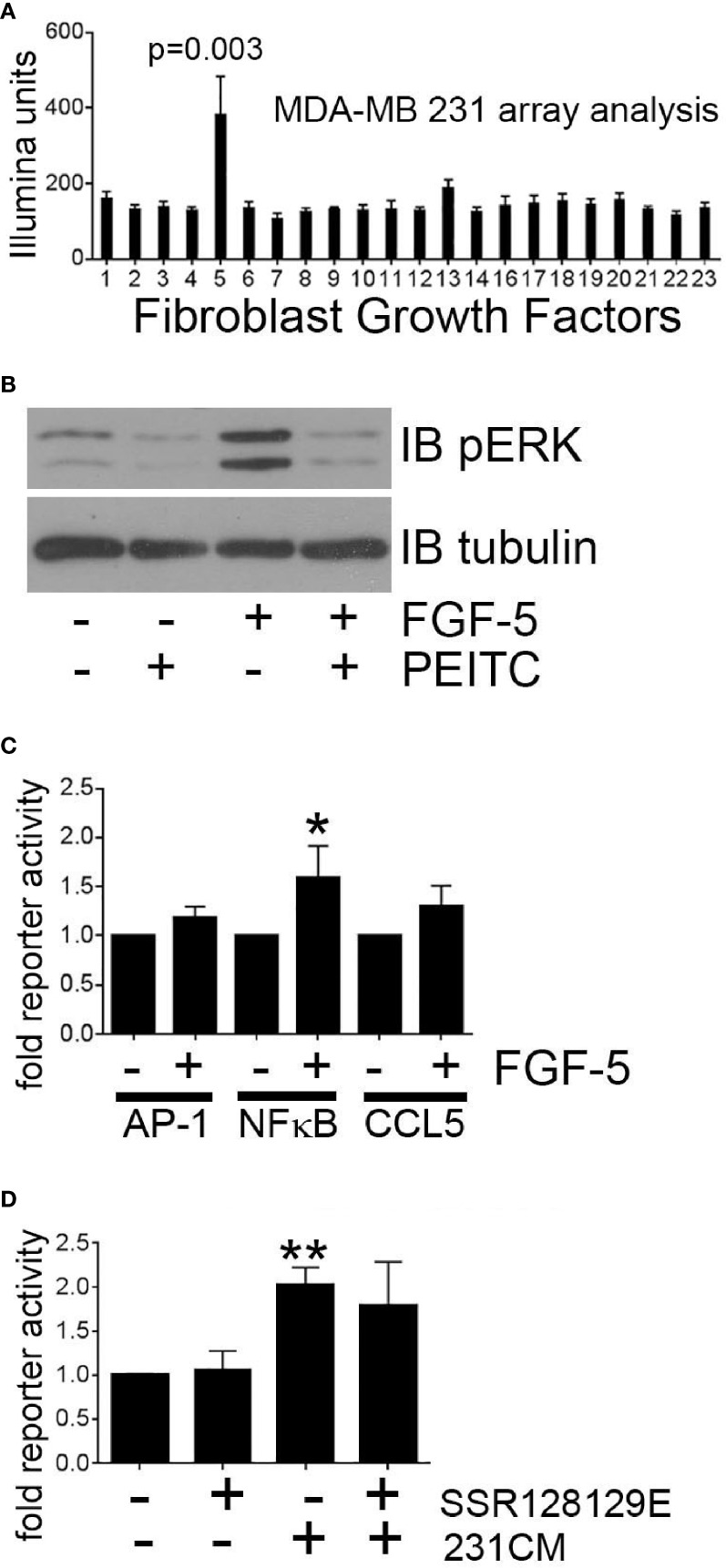
Breast tumor cell-derived FGF-5 activates MEKK1 in mammary fibroblasts. Analysis of MEKK1 signaling induced by MDA-MB 231-derived FGF-5. **(A)** Graphical representation of relative expression of FGF genes expressed in cultured MDA-MB 231 cells as detected by Illumina expression array analysis. X axis label indicates FGF family member assigned to each column (column 5 = FGF-5). **(B)** Phospho-ERK1/2 MAPK immunoblot analysis of lysates extracted from mammary fibroblast pre-treated for 1 h with vehicle or PEITC (0.3 µM) prior to stimulation with FGF-5 (25 ng/ml). An anti-tubulin immunoblot is displayed as a loading control. **(C)** Luciferase reporter analysis of mammary fibroblasts transfected with reporter plasmids wherein luciferase expression is driven by either the CCL5 promoter or response elements that bind AP-1 or NFκB. **(D)** CCL5 reporter analysis of mammary fibroblasts treated with FGFR1 inhibitor SSR128129E (0.3 µM) prior to an 8 h stimulation with culture media conditioned by MDA-MB 231 cells (231CM). The data represented in graphs are derived from three independent experiments. *p < 0.05, **p < 0.01 against control by t-test.

Breast tumor cells have been reported to secrete multiple factors that may activate mammary fibroblast signaling. To identify tumor cell-derived secreted factors that induce MEKK1-dependent CCL5 expression, we performed expression analysis of mRNA extracted from MDA-MB 231 cells (three replicates) and observed that of the fibroblast growth factor (FGF) family of secreted factors, FGF-5 alone was highly expressed (**Figure 6A**). FGF-5 is a ligand for the receptor tyrosine kinase FGFR1 (39), and although FGFR1 ligation initiates cell signaling that regulates cell function, our investigation did not reveal any published study linking FGF-5 activation to MEKK1. Active MEKK1 phosphorylates and activates kinases of the MEK family that subsequently phosphorylate the activation loop of JNK and ERK1/2 MAPKs (30), so we utilized immunoblot analysis with phospho-specific ERK1/2 (T202/Y204) and JNK (T183/Y185) antibodies to determine whether FGF-5 activates MAPK signaling. We observed that exposure to recombinant FGF-5 had little effect on JNK phosphorylation (not shown), but consistently induced ERK1/2 phosphorylation that was reversed by the MEKK1 inhibitor PEITC (**Figure 6B**). Finally, we determined whether FGF-5-induced signaling induces CCL5 expression by performing CCL5 reporter assays in mammary fibroblasts treated with recombinant FGF-5. Since we had found that CCL5 expression was sensitive to inhibition of NFκB and AP-1, we also performed reporter assays to determine whether FGF-5 promotes activation of these CCL5-regulating transcription factors. As predicted, we found that FGF-5 significantly (53%) activated NFκB reporter activity, and consistently activated both the CCL5 (30%) and AP-1 (16%) reporter assays (**Figure 6C**). Finally, we examined the importance of tumor-derived FGF-5 to CCL5 expression in mammary fibroblasts. FGF-5 is a ligand for FGFR1 and FGFR2, and to determine whether inhibition of FGFR1 activation alone was responsible for CCL5 reporter activity induced in mammary fibroblasts by tumor-derived factors, we pre-treated mammary fibroblasts with the selective FGFR1 inhibitor SSR128129E prior to stimulation with breast cancer cell-conditioned media (**Figure 6D**). We observed only a modest partial inhibition of CCL5 reporter activity, suggesting that FGFR1-independent signaling plays a major role in tumor cell-induced CCL5 expression in mammary fibroblasts.

## Discussion

For cancers to metastasize to secondary tissues and organs, cancer cells must first migrate from the primary lesion and invade into the surrounding stroma. One possible mechanism driving cancer cell expansion into surrounding tissues and distant organs would be for cancer cells to migrate toward a source of chemoattractant, such as organs or tissue environments that produce migration-inducing chemoattractants. Soluble chemoattractants such as chemokines drive directed cell migration, and breast cancer cells that express chemokine receptors can migrate in the direction of increasing concentrations of cognate chemokine ligands present in the tumor microenvironment. The chemokine ligand CCL5/RANTES drives directed migration of cells that express the chemokine receptors activated by CCL5, including the cell surface receptor CCR5. While the importance of CCL5 in driving leukocyte migration has long been known, CCL5 may well induce chemotaxis in other cells that similarly express one or more of the CCL5 receptors.

**Figure 7:**
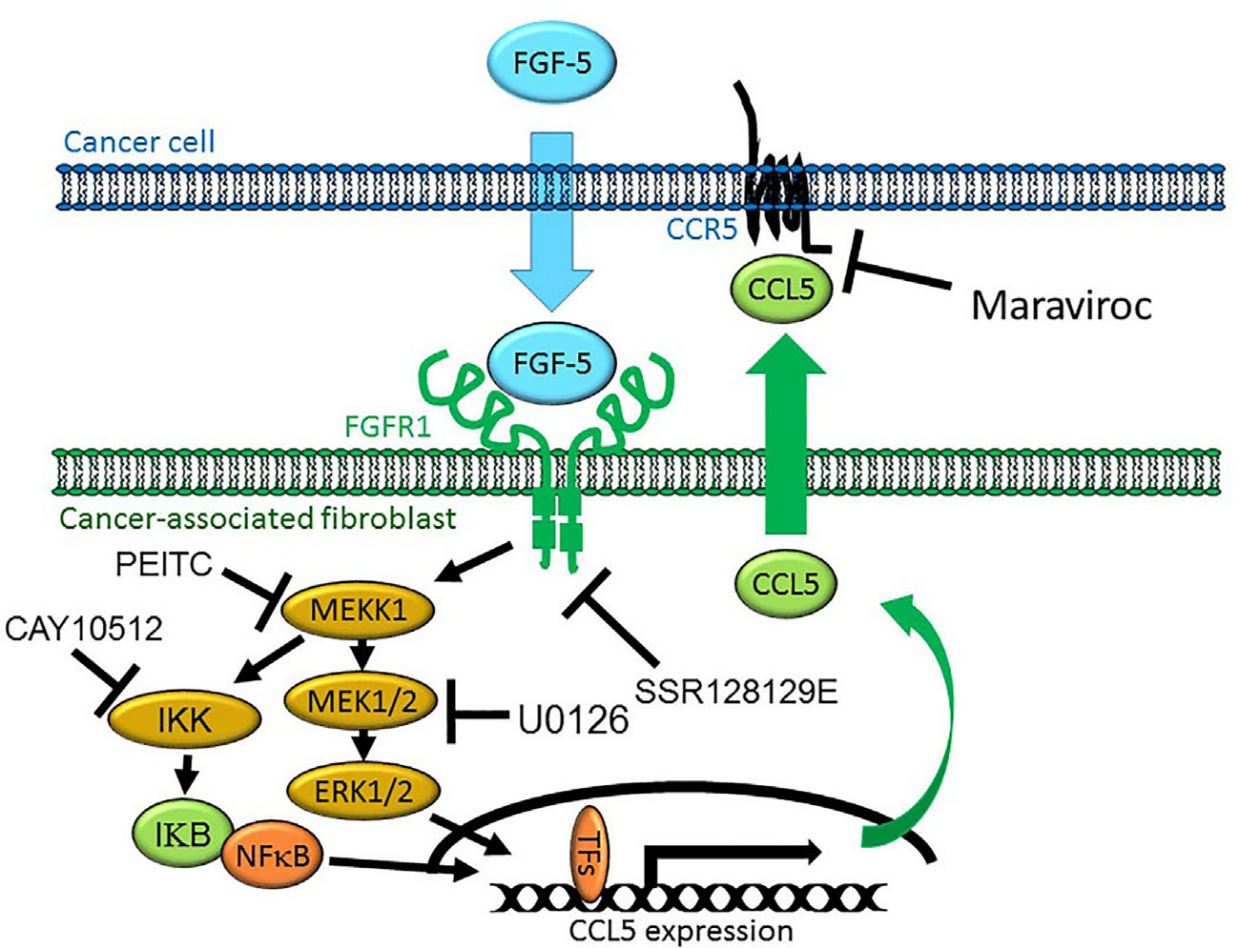
Model of MEKK1-Ccl5 expression induced by paracrine interaction between breast cancer cells and mammary stroma. MDA-MB 231 breast cancer cells express and secrete FGF-5 in the tumor microenvironment, inducing expression of mammary fibroblast-derived CCL5 that promotes tumor cell migration. FGF-5 binds to FGFR1 proteins on the surface of mammary fibroblasts, triggering receptor tyrosine kinase activity that induces MEKK1 activation and signaling that leads to the phosphorylation of transcription factors, binding of an active transcriptional complex to the CCL5 promoter and CCL5 expression.

Our data would suggest that MEKK1 is an important regulator of chemokine expression in fibroblasts within the breast stroma tissue that serve as a major source of chemokines that induce chemotaxis of cancer cells (**Figure 7**), possibly contributing to cancer cell migration to distant tissues and organs from the primary tumor. It is important to note that our model represents a part of a very complex cellular system, and does not exclude the possibility that other stroma cell types may contribute to the overall protein concentration of stroma-derived CCL5 within tumors. We recognize that breast cancer cells may produce secreted factors other than FGF-5 that induce expression of CCL5. Furthermore, fibroblasts and other stroma cells localized in additional non-tumor tissues could release chemokines that provide a chemoattractant stimulus for breast tumor cells to localize to those tissue, thereby providing a means by which breast tumor cells home to the sites of secondary tumor formation and eventual metastases.

CCR5 function is well described in leukocytes such as lymphocytes, neutrophils and macrophages. CCR5 binding to chemokine ligands secreted at sites of inflammation contributes to the ability of leukocytes to detect and migrate to inflamed tissues in order to promote a functional immune response. While it is an important component a functional immune system, CCR5 also represents an avenue by which the immune system is compromised by viral infection. HIV utilizes CCR5 to bind and infect target lymphocytes, and discovery of the HIV-CCR5 interaction led to the development of Maraviroc as an FDA-approved HIV therapeutic agent. It is reasonable to postulate that nonimmune cells that express CCR5 may migrate in response to a concentration gradient of CCL5, and thus investigation of CCR5 blockers as breast cancer therapeutic agents is in its early stages, and is bolstered by data from animal models of tumor progression showing promise as anti-metastasis agents (12). We recognize that, as our data identify MEKK1 as a regulator of multiple chemokines, selective MEKK1 inhibition may have functional consequences that far exceed the impact of CCR5 inhibition alone (e.g. immune function) that will influence the utility of MEKK1 inhibitors as cancer therapeutic agents. We have chosen to focus our functional assays on CCL5 in part because of the availability of Maraviroc as an FDA-approved HIV therapeutic that we would propose may have the potential to be repurposed for cancer treatment. We realize that much work remains to be done to investigate the importance of the other MEKK1-dependent chemokines identified in this study.

In this study, we have found that mammary fibroblasts express chemokines that promote immune cell directed migration. We demonstrated that CCL5 expression is strongly promoted by MEKK1 kinase activity, and that ablation of MEKK1 kinase activity, either *via* gene knockout, chemical inhibition of MEKK1, or transfection of dominant negative MEKK1, greatly reduces CCL5 expression. We previously reported that MEKK1 signaling is required for expression of proteases that are transcriptionally controlled by AP-1 and promote tumor invasiveness (31), so our discovery that MEKK1 controls chemokine gene expression in breast stroma cells strongly supports a model of the breast tumor microenvironment in which MEKK1-dependent gene expression in stroma cells can promote breast cancer cell migration. When combined with our previous reports, a paradigm emerges wherein MEKK1 controls gene expression in both malignant cancer cells and non-cancer stroma cells, and MEKK1-dependent genes in both cell types promote invasion and metastasis.

MEKK1 belongs to a family (MAP3K) of more than twenty different protein kinases, with many exhibiting similar substrate preferences. For example, six of these kinases can activate ERK1/2 signaling, whereas at least fourteen drive JNK activation (30). We maintain that one evolutionary explanation for the abundance of MAPK regulators may be that many cellular functions require carefully modulated MAPK activity, and that response to distinct signals by different MAP3K proteins promotes specific functional outcomes. If several related proteins have similar MAPK regulatory capabilities, why is MEKK1 essential to CCL5 expression? One function that distinguishes MEKK1 from some other MAP3K is that it can activate IKB kinases to promote NFκB activity. Combined with the capacity to control AP-1 activity by promoting both MAPK signaling and ubiquitin ligase activity, MEKK1 has the unique capacity to serve as a transcriptional regulator of both AP-1 and NFκB-dependent gene expression. Why does ablation of MEKK1 result in a marked reduction of CCL5 expression? We propose that the ability of MEKK1 to integrate signals arising from environmental stimuli that activate both AP-1 and NFκB activity may be necessary for expression of genes like CCL5 that require both NFκB and MEKK1-dependent AP-1 transcriptional complexes.

## Conclusions

In conclusion, we provide evidence that MEKK1-dependent CCL5 expression in mammary fibroblasts can induce a functional response in breast tumor cells that is a key requirement for breast tumor metastasis to occur. When considering our previous reports that MEKK1 regulating breast tumor cell function, and that MEKK1-deficient mice are significantly protected from breast tumor metastasis, we conclude that MEKK1 activity in both malignant breast cancer cells and non-cancer stroma cells drive the creation of a breast tumor environment that favors metastasis. Chemokines have been proposed to be potential breast cancer therapeutic targets, and our work provides evidence to support the contention that pharmacologic inhibition of MEKK1 may be one approach to reduce expression of chemokines and thereby alter the tumor microenvironment that promotes metastasis. At present, we would postulate that CCR5 competitive inhibitors such as Maraviroc should be investigated in future translational research as potential anti-metastasis therapeutics.

## Data Availability Statement

The datasets presented in this study can be found in online repositories. The names of the repository/repositories and accession number(s) can be found below: NCBI [accession: GSE167130].

## Author Contributions

SG contributed to the design, and executed and analyzed all the experiments in this research work. MR executed and analyzed all the experiments in this research work. BC contributed to the design, and executed and analyzed all the experiments in this research work. All authors contributed to the article and approved the submitted version.

## Funding

This work was supported by the American Cancer Society, Illinois Div. [Grant 160485 (to BC)], by the Department of Defense [US DoD, CDRM W81XWH-20-1-046 (to SG)], and the Christl Burgess Memorial Fund for Ovarian Cancer Research (to SG).

## Conflict of Interest

The authors declare that the research was conducted in the absence of any commercial or financial relationships that could be construed as a potential conflict of interest.
